# Intradural extramedullary capillary hemangioma with intramedullary component: A case series

**DOI:** 10.1097/MD.0000000000029862

**Published:** 2022-07-29

**Authors:** Zhen Zhao, Jin Zheng, Yingchun Zhou

**Affiliations:** a Department of Neurosurgery, Union Hospital, Tongji Medical College, Huazhong University of Science and Technology, Wuhan 430022, China.

**Keywords:** case report, intramedullary, intramedullary and extramedullary, spinal cord capillary hemangioma

## Abstract

**Rationale:**

Capillary hemangioma in the spinal cord is an exceedingly rare entity that is liable to be misdiagnosed. To summarize the clinical presentation, radiological characteristics, and pathological features of capillary hemangioma in the spinal cord and to share our experience for the diagnosis and treatment of intradural extramedullary capillary hemangioma.

**Patient concerns:**

Three patients underwent surgical treatment at our hospital between January 2020 and August 2020. All patients were male[median age at presentation: 57 years (range: 56–60)]. Two patients were experiencing pain and numbness in the lower back, and 1 patient was experiencing numbness and weakness in the left lower limb. The duration of symptoms ranged from 1 to 5 months.

**Diagnosis:**

All patients were diagnosed with spinal cord capillary hemangioma after treatment. All lesions were in an intradural extramedullary location and involved spinal cord components. Two patients had lesions in thoracic segments (T8, Th9-10), and 1 patient had a lesion in lumbar segment (L1).

**Interventions:**

All patients underwent microscopic laminectomy and complete resection of the extramedullary and intramedullary components of the spinal cord capillary hemangiomas.

**Outcomes:**

Postoperatively, all patients experienced transient numbness and pain in the lower limbs, which gradually decreased over time. None of the patients experienced bleeding, severe numbness or pain, or recurrence of symptoms at 3-month follow-up.

**Conclusion:**

Intradural extramedullary capillary hemangioma has unique morphological characteristics. Gross-total resection of the extramedullary and intramedullary components of spinal cord capillary hemangioma is recommended for patients with symptoms of spinal cord compression. Careful preoperative imaging and intraoperative decision-making may help avoid residual lesions or reoperation.

## 1. Introduction

Capillary hemangioma is a ubiquitous benign vascular malformation caused by the proliferation of hemangioblasts. Capillary hemangiomas are typically observed in neonates at or shortly after birth.^[1–4]^ Capillary hemangiomas are usually found in the skin, soft tissues, and bones, but may rarely occur in the central nervous system. Capillary hemangiomas in the spinal cord are exceedingly rare.^[5,6]^ Some reports have described intradural extramedullary capillary hemangiomas (not involving the spinal cord) or epidural capillary hemangiomas. A few reports have described cases of spinal cord capillary hemangiomas with extramedullary and intramedullary components in whom the presenting signs and symptoms were caused by compression or invasion of the spinal cord by the lesion.^[7,8]^ Here, we report 3 cases of spinal cord capillary hemangioma with extramedullary and intramedullary components in the thoracic and lumber spine and summarize the associated clinical presentation, radiological characteristics, and pathological features.

## 2. Case presentation

### 2.1. Patients

This study included 3 patients who were diagnosed with spinal cord capillary hemangioma and underwent surgical treatment at our hospital between January 2020 and August 2020. All patients were male [median age at presentation: 57 years (range: 56–60)]. The study protocol complied with the principles enshrined in the Helsinki Declaration and was approved by the Institutional Review Board of the Union Hospital. Written informed consent was obtained from all patients. Patient data, including sex, age, symptoms, radiological characteristics, treatment interventions, and pathological results were retrieved and retrospectively reviewed.

### 2.2. Case 1

**Figure 1.**
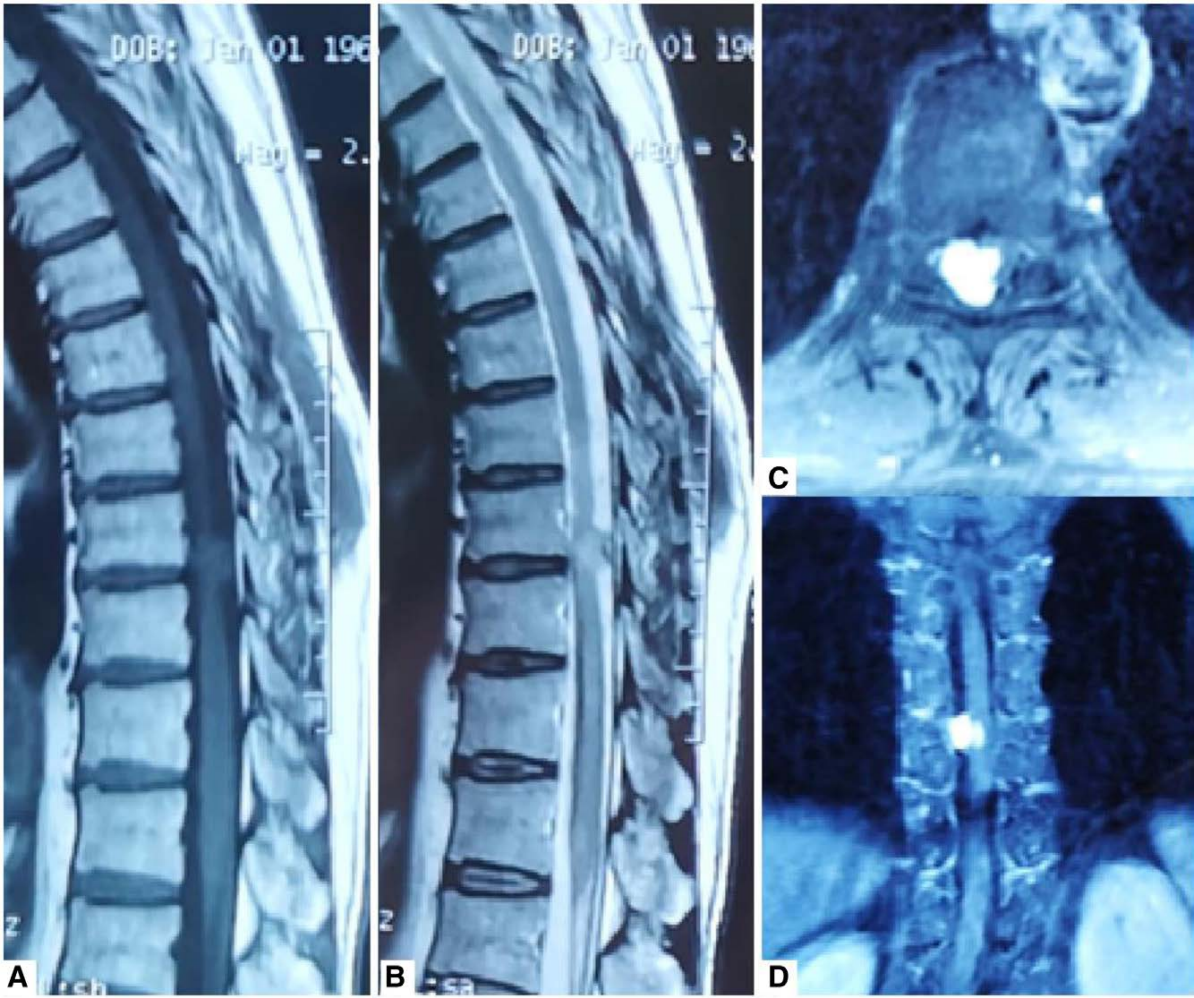
A 56-year-old man presented with a history of progressive low-back pain and numbness of the right lower limb for 1 month. Preoperative MRI. (A) T1WI sequence shows an isointense lesion; (B) T2WI sequence shows a hyperintense lesion with spinal cord edema; (C and D) Axial and coronal CE-T1WI show a homogeneous enhanced mass compressing the spinal cord.

The patient was a 56-year-old man who presented with the chief complaint of progressive lower back pain and numbness of the right lower limb for 1 month. These symptoms were not alleviated by acetaminophen. Physical examination showed normal muscle strength in both upper and lower limbs, normal deep and superficial reflexes, but an abnormal sensation in the right lower limbs. MRI revealed a large intradural lesion at T9 to T10, with a high signal on T2-weighted images, homogenous enhancement, and spinal cord edema (Fig. 1). Based on the imaging characteristics, the preoperative diagnosis was hemangioblastoma (Table 1 and Fig. 1).

**Table 1:** Characteristics of the 3 patients with spinal cord capillary hemangioma.

							MRI features					Recurrence
Case No.	Sex	Age, y	Symptom	Duration of Illness	Lesion location	Preoperative diagnosis	T1WI	T2WI	+GD	Resection rate	Follow-up, mo	
1	M	56	MBP, LBP, right LLN	1 months	T9-10 (EM and IM)	Hemangio-blastomas	ISO	HY	HE	GTR	3	No
2	M	56	Left LLNand LLK	5 months	T8 (EM and IM)	Hemangio-blastomas	ISO	HY	HE	GTR	3	No
3	M	60	LBP, left LLN	4 months	L1 (EM and IM)	Schwannoma	ISO	HY	HE	GTR	3	No

**Figure 2.**
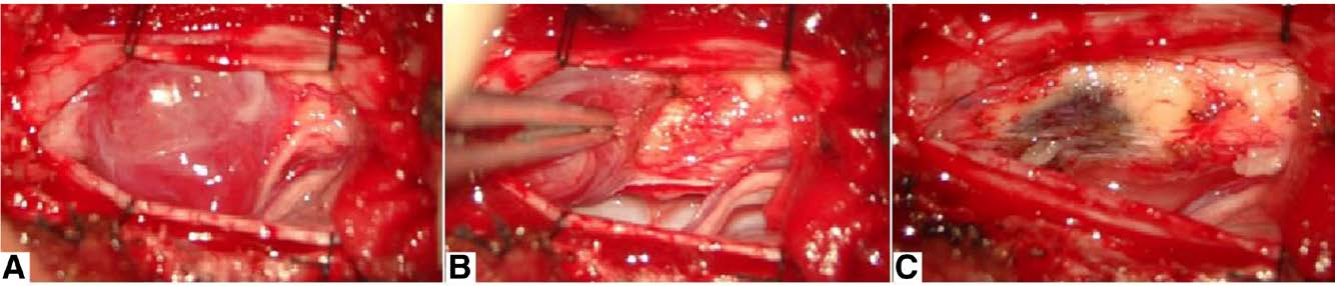
Surgical procedure. (A) Subarachnoid tumor with rich-blood supply; (B) the mass was dissected along the margin; (C) abnormal brown tissue on the spinal cord, with a distinct boundary at the pia mater spinalis.

**Figure 3.**
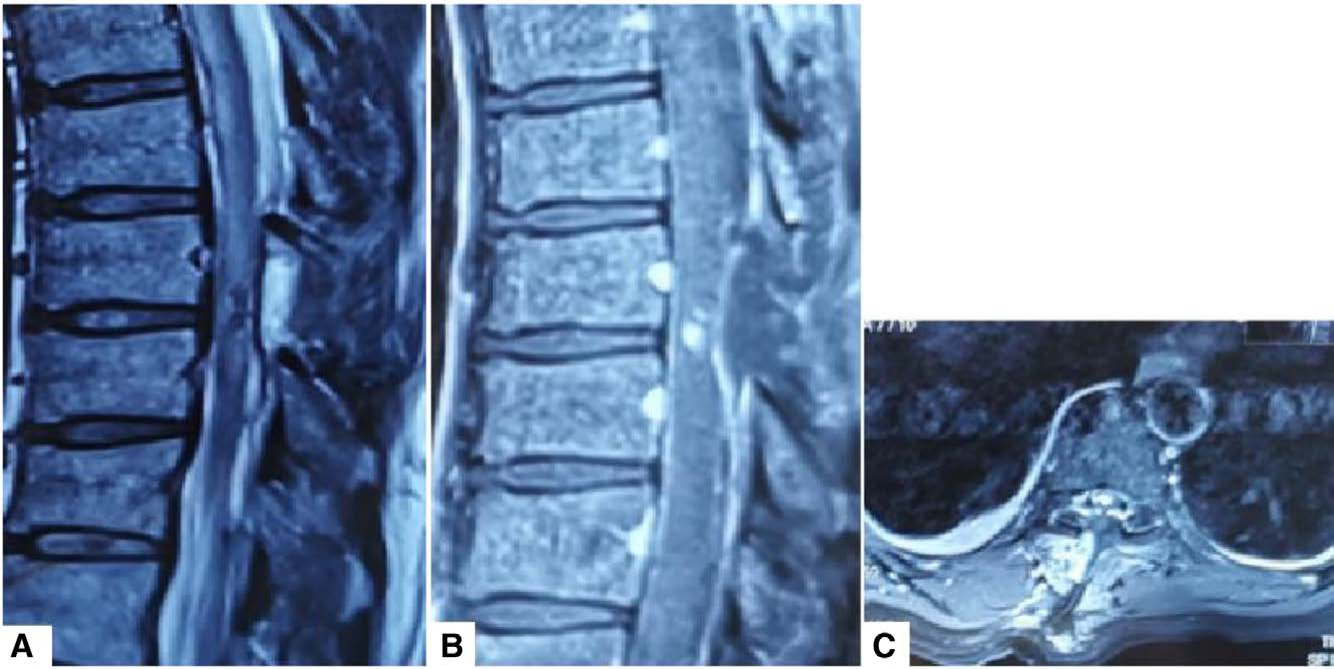
Postoperative MRI. Postoperative MRI showing a residual intramedullary lesion. MRI = magnetic resonance imaging.

**Figure 4.**
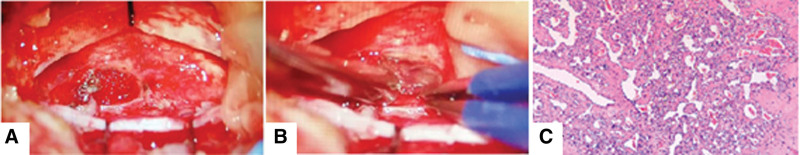
The second surgical procedure. (A) The pia mater was incised to reveal the intramedullary lesion; (B) the intramedullary lesion was completely resected. (C) Capillary hemangioma accompanied with active cell growth (HE stained, 100× magnification).

Microscopic laminectomy was performed under general anesthesia with the patient placed in the prone position. A posterior midline incision was made through the skin and paravertebral muscles. After opening the dura mater, a purple-red mass with clear boundaries, soft texture, and abundant blood supply was observed in the subarachnoid space (Fig. 2A). Although the lesion was attached to the spinal cord, it could be completely removed (Fig. 2B); however, there was a residual abnormal brown area on the spinal cord, with a distinct boundary at the pia mater spinalis (Fig. 2C). It was unclear if this brown area was another lesion or an abnormal blood vessel caused by the original lesion. The presence of intramedullary components was suspected but not investigated further during surgery to avoid unnecessary incision of the medullary tissue, which may have caused neurological deficit. The operation was terminated, and further imaging was performed to inform clinical decision-making. Postoperative MRI showed a lesion in the spinal cord (Fig. 3). A second operation was performed on the subsequent day, during which a tumor was removed from the spinal cord after incising the pia mater spinalis (Fig. 4A and B).

On gross examination, the resected lesions were gray or red-brown. Microscopic examination showed expansion of small blood vessels with a uniform lining of endothelial cells in the spinal cord interstitium. The blood vessels were separated by fibrous tissue that did not contain stromal cells. On immunohistochemical examination, the blood vessels stained positive for the capillary markers CD31, CD34, and ERG, and negative for smooth muscle agonist protein, epithelial membrane antigen, glial fibrillary acidic protein, inhibin, S100, D2-40, and CD68. The Ki67 cell proliferation index was <20%. Histological and immunohistochemical staining confirmed the diagnosis of capillary hemangioma (Fig. 4C).

After the second operation, the patient experienced transient numbness and pain in the lower limbs. However, there was no bleeding from the lesion. There was no severe numbness or pain, or recurrence of symptoms during 3 months of follow-up.

### 2.3. Case 2

**Figure 5.**
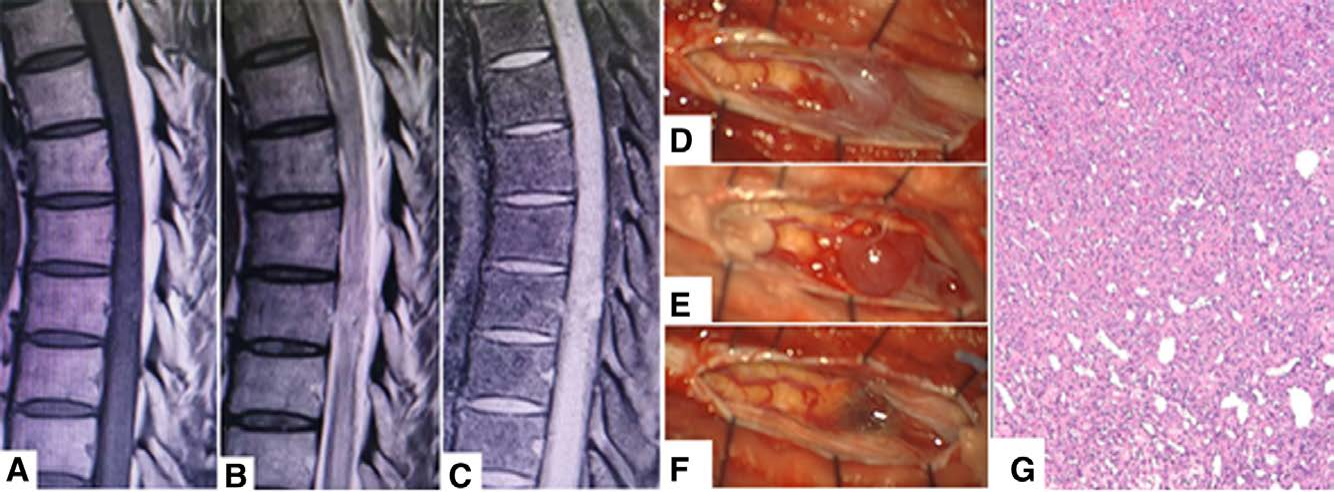
A 56-year-old man presented with a 5-month history of left lower limb numbness and lower limb weakness. (A–C) Preoperative MRI. The lesion is at T8 level. (D) The dura was opened; (E) extramedullary components; (F) an abnormal area is seen on the spinal cord, with a distinct boundary at the pia mater spinalis. (G) Photomicrograph of the tumor specimen (HE stained, 100× magnification). MRI = magnetic resonance imaging.

Case 2 was a 56-year-old man who presented with a history of left lower limb numbness and lower limb weakness for 5 months. MRI spine revealed a lesion at T8 level with both extramedullary and intramedullary components. The lesion exhibited a high signal on T2-weighted images, homogenous enhancement, and spinal cord edema (Fig. 5A–C). The preoperative diagnosis was hemangioblastoma as in Case 1, with similar MRI features (Table 1).

The patient underwent microscopic laminectomy. Based on our experience of the previous patient, on finding an abnormal brown area on the spinal cord, we decided to continue the surgery, invade the spinal cord tissue and remove the intramedullary portion of the tumor (Fig. 5D–F). The lesion was diagnosed as spinal cord capillary hemangioma based on histopathological examination of surgical specimen (Fig. 5G).

The patient experienced a mild aggravation of the preoperative symptoms for the first 2 weeks after operation, which gradually improved later. Routine follow-up including repeat spinal MRI was performed 3 months after discharge. Clinical and neuroimaging findings were compared to baseline presentation. There was no severe pain, paralysis, or recurrence of symptoms in the 3 months of follow-up.

### 2.4. Case 3

**Figure 6.**
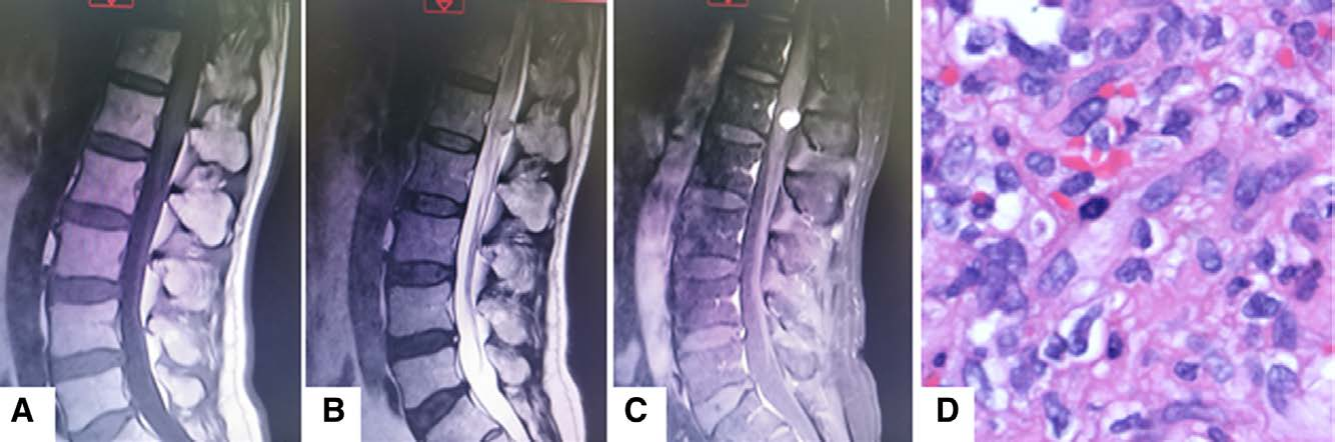
A 60-year-old man presented with left back pain and left lower limb numbness for 4 months. (A–C) Preoperative MRI. The lesion is seen at L1. (D) Lobulated capillary hemangioma accompanied with active cell growth (HE stained, 100× magnification). MRI = magnetic resonance imaging.

Case 3 was a 60-year-old man who complained of left back pain and left lower limb numbness for 4 months. preoperative spinal MRI showed a mass at level L1 which appeared hyperintense on T2-weighted images and isointense on T1-weighted images, and showed homogenous enhancement (Fig. 6A–C).

According to the patient’s medical records and MR images, the lesion was diagnosed as an intradural–extramedullary Schwannoma at level L1 (Table 1). The patient underwent microscopic laminectomy soon. Intraoperatively, we found not only the intradural part of the tumor, but also an intramedullary portion with similar appearance. Unfortunately, the operation imaging data are missing, but a diagnosis of spinal cord capillary hemangioma was confirmed by histopathological examination of the surgical specimen (Fig. 6D). Postoperatively, the patient experienced mild aggravation of lower limb numbness and developed urination disturbances in the first postoperative week. His back pain resolved completely. The patient has managed to return to normal life at 3-month follow-up, with no severe pain or urination disturbance, or recurrence of symptoms.

## 3. Discussion

Currently, no >100 cases of spinal cord capillary hemangioma have been reported worldwide, most of which were extramedullary capillary hemangioma with no intramedullary components. A literature search on PubMed for studies describing spinal cord capillary hemangioma with extramedullary and intramedullary components retrieved 6 articles containing reports of 10 patients.^[7–12]^ The demographic, imaging, and clinical characteristics of these patients are summarized in Table 2. Among the patients described in the present and previous reports, the majority were male (male:female = 12:1); the age range of patients was 43 to 80 years, with most patients aged between 40 and 60 years. Lesions occurred in the thoracic or lumbar spinal cord, and mainly involved 1 or 2 vertebral segments. The most common symptoms included middle-back or lower-back pain, lower limb pain and numbness, lower limb weakness, and paresthesias, which were experienced for 1 month to 1 year. These symptoms are similar to the clinical manifestations associated with disc herniation or foraminal stenosis; therefore, spinal MRI is essential for differential diagnosis.

**Table 2:** Previous reports of spinal cord capillary hemangiomas with extramedullary and intramedullary components.

								MRI features					Recurrence
Study	Country	Age (y)	Gender	Symptom	Duration of illness	Lesion location	Preoperative diagnosis	T1WI	T2WI	+GD	Resection rate	Follow-up	
Gonzalez et al^[7]^	Canada	59	M	Progressive left LLN	7 mo	T7-8	Hypervascular neoplasm	NR	NR	NR	NR	3 mo	No
Wu et al^[9]^	China	59	M	MBP; bilateral LLN & LLK	1 y	T3-4	Schwannomas	Irregular ISO	Mild HY	HE	STR	24 mo	Reoperation
Kasukurthi et al^[8]^	USA	47	M	MBP; hyperesthesia of lower limbs; gait difficulties	Several months	T3	NR	NR	NR	HE	NR	NR	No
Kelleher et al^[10]^	England	57	M	Progressive thoracic pain; bilateral LLK	7 mo	T9-10	NR	ISO	HY	Strong enhancement	GTR	3 mo	No
Abe et al^[11]^	Japan	51	M	LLK	1 mo	T11	NR	Mild HY	HY	Strong HE	NR	NR	No
		64	M	LLN	2 mo	T7	NR	Mild HY	HY	Strong HE	NR	NR	No
		71	M	LLN	3 mo	T11	NR	Mild HY	HY	Strong HE	NR	NR	No
		80	M	LLN	4 mo	T9	NR	Mild HY	HY	Strong HE	NR	NR	No
		43	M	LLN	2 mo	T7	NR	Mild HY	HY	Strong HE	NR	NR	No
Shin et al^[12]^	Korea	66	F	LBP	8 mo	T8-9	NR	ISO	HY	Strong HE	GTR	6 mo	No

On preoperative spinal cord MRI, spinal cord capillary hemangioma with extramedullary and intramedullary components appeared isointense compared to the spinal cord on T1-weighted images, and hyperintense on T2-weighted images. The lesions showed homogeneous strong enhancement on contrast-enhanced T1-weighted sequences. The symptoms and imaging features of spinal cord capillary hemangioma were nonspecific; therefore, spinal cord capillary hemangioma may be misdiagnosed as other tumors of the spinal cord, including intramedullary ependymoma, hemangioblastoma, extramedullary schwannoma, cavernous vascular malformations, or tumor metastases. In the present case series, the preoperative diagnosis was hemangioblastoma in 2 patients and schwannoma in 1 patient. In previous reports, the most common preoperative misdiagnosis of spinal cord capillary hemangioma was schwannoma.

We recommend a comprehensive preoperative evaluation of patients with spinal cord capillary hemangioma and symptoms of spinal cord compression to avoid permanent structural and functional injury of the spinal cord. All patients in the present case series underwent laminectomy. In the first case, the extramedullary component was easily located and completely resected; however, there was a residual abnormal brown area on the spinal cord, with a distinct boundary at the pia mater spinalis. It was unclear whether the brown area was a sign of spinal cord compression or invasion. Considering that pia mater spinalis had no defects, and incision of the spinal cord may cause catastrophic complications, surgery was ended without exploring the spinal cord. Postoperative MRI confirmed the need for a second operation, during which a tumor was removed after incising the spinal cord. Based on this experience, the next 2 patients underwent a single surgery during which the extramedullary and intramedullary components of the spinal cord capillary hemangiomas were excised. None of the patients in our series experienced bleeding from the lesion, severe numbness or pain, or recurrent symptoms within 3 months of follow-up. To avoid recurrence and further surgeries, we recommend complete resection of spinal cord capillary hemangioma. In a previous report, a patient who underwent subtotal removal of an intramedullary spinal cord capillary hemangioma required a second surgery due to enlargement of the residual lesion.^[9]^ Some studies have suggested that glucocorticoids can inhibit the growth of capillary hemangioma, and specifically, spinal cord capillary hemangioma.^[10,11]^ However, it is currently unknown whether treatment of spinal cord capillary hemangioma with corticosteroids offers a therapeutic advantage over the surgical approach.

## 4. Conclusion

Spinal cord capillary hemangioma should be considered in patients presenting with spinal cord tumors with intramedullary components. Early and complete resection can relieve the edema caused by spinal cord compression and avoid permanent structural and functional damage to the spinal cord. Intradural extramedullary capillary hemangioma has unique morphological characteristics, but its appearance can be misleading. Intradural extramedullary capillary hemangioma may be separated into 2 parts by the pia mater spinalis, and a residual lesion may be left after surgery. Careful preoperative imaging evaluation and intraoperative assessments are required to manage intradural extramedullary capillary hemangioma. Our clinical experience may inform clinical decision-making and help avoid the need for a second operation in patients with spinal cord capillary hemangioma.

## Author contributions

Conceptualization: Y.Z.

Data curation: J.Z., Z.Z.

Formal analysis: Z.Z., J.Z.

Investigation: J.Z.

Methodology: Y.Z.

Supervision: Y.Z.

Validation: Y.Z., J.Z.

Visualization: Y.Z., J.Z.

Writing - original draft: Z.Z.

Writing - review & editing: J.Z., Y.Z.
